# Differences in community structure of gastrointestinal tract between *Helicobacter pylori* positive patients and negative patients with gastric cancer

**DOI:** 10.7150/jca.69873

**Published:** 2022-03-28

**Authors:** Jianing Ding, Yan-gao Man, Xiaorong Deng, Tingtao Chen

**Affiliations:** 1 Department of Gastrointestinal Surgery, The Second Affiliated Hospital of Nanchang University, Nanchang, Jiangxi 330006, PR China.; 2 Queen Mary School, Nanchang University, Nanchang, Jiangxi 330031, P.R. China, Nanchang University, Nanchang 330031, PR China.; 3 Department of Pathology, Hackensack University Medical Center, 30 Prospec Avenue, Hackensack, NJ 07601, USA.; 4 National Engineering Research Center for Bioengineering Drugs and the Technologies, Institute of Translational Medicine, Nanchang University, Nanchang 330031, PR China.

**Keywords:** *H. pylori*, gastric cancer, gastrointestinal microbiota, gastrointestinal microbiota disorder

## Abstract

Gastric cancer is one of the most severe cancers, while the relationship between* Helicobacter pylori* (*H. pylori*) and gastric cancer are still in dispute, and little work has been done to explore the microbial diversity between *H. pylori* positive patients and negative patients. In the present work, a total of 43 gastric cancer patients and 10 healthy 53 participants were enrolled to compare the microbial differences in community structure in gastrointestinal tract between *H. pylori* positive patients and negative patients with gastric cancer. Our results indicated that the abundance and diversity of gastrointestinal microbiota was slight lower in gastric cancer patients than that in healthy participants especially in intestine, while the abundance of some potential pathogens, e.g. *Streptococcus*, *Lactobacillus*, *Akkermansia* and *Halomones* were higher in *H. pylori* positive patients than *H. pylori* negative patients. Therefore, our work suggests the various microbial diversity between *H. pylori* positive patients and *H. pylori* negative patients with gastric cancer, which contribute to deepen the understanding of the role of *H. pylori* in gastric carcinogenesis and progression.

## Introduction

Gastric cancer is a serious malignancy worldwide, with about one million new cases diagnosed each year, contributing to 783,000 deaths in 2018, and an average 5-year survival rate of less than 20%, which imposes a huge medical and economic burden on society 1-3. Until now, the understanding of gastric cancer is not thorough enough, and there are issues such as difficulty in early detection, high recurrence rate, rapid development and unclear pathogenesis 4. Thus, exploring the pathogenesis and pathogenic factors is of great significance to the prevention and treatment of gastric cancer 5, 6.

At present, widely recognized carcinogenic factors include high salt-preserved diet, chemical exposure, smoking, pernicious anaemia and stomach related diseases 6. With the development of high-throughput sequencing technology, there is a growing body of opinion suggesting that the gastrointestinal microbiota is closely related to the development and progression of gastric cancers 7-9. For example, Castaño-Rodríguez investigated the alteration of gastrointestinal microbiota composition and main species during the development of gastric cancer through a clinical cohort study and discovered the patients with gastric cancer had an imbalance in the gastrointestinal microbiota, manifested by an increase in *Lactobacillus* and *Lactococcus* compared with control group which could overproduce lactic acid to contribute the growth and angiogenesis of gastric cancer as energy 10, 11. Moreover, Soon-Kyeong Kwon et al. found that implanting the microbiota from the stomach of gastric cancer patients to germ free mice caused premalignant lesions including the losing of parietal cells and exhibiting inflammation, and that using the probiotics *L. plantarum*, *L. rhamnosus*, *L. acidophilus* and *Bifidobacterium* as adjuvant therapy could significantly lower postoperative inflammation, increase immunity and correct disturbances in the gastric microbiota 12, 13.

In history, the stomach was considered sterile due to its strong acid environment, then Marshall and Warren discovered the *H. pylori* and confirmed its role in gastric diseases including gastritis and gastric ulcer 14, 15. In 1995, a total of 3,365 participants were enrolled in the study, and over a 15-year follow-up, eradication of *H. pylori* resulted in a 39% reduction in the incidence of gastric cancer compared to the control group 16. Similarly, reductions in *H. pylori* infection over the past 50 years have led to a decline in the incidence of gastric cancer in Western countries 17, 18. However, some studies indicated that the elimination of *H. pylori* could not reduce the incidence of all types of gastric cancer such as cardia cancer, whose incidence remains stable and even increases in certain countries such as Europe, Japan and North America 19, and that statistics showed that only 3% of those infected will develop gastric cancer and that the vast majority of those infected with *H. pylori* live in symbiosis without gastric cancer 20. Thus, until now, the relationship between *H. pylori* and gastric cancer has been the focus of debate.

In the present study, a total of 53 volunteers including 23 *H. pylori* infection positive gastric cancer cases, 20 *H. pylori* infection negative gastric cancer cases and 10 normal healthy participants were enrolled, and their alteration of gastrointestinal microbiota and intestinal microbiota were tested using 16S rRNA sequence. Our aim is to reveal the correlation between gastric cancer and *H. pylori* infection and to provide positive suggestions for the treatment of gastric cancer.

## Materials and Methods

### Study Design and Ethical Approval

The randomized clinical samples were collected from the Second Affiliated Hospital of Nanchang University and Second Affiliated Hospital of Shandong University from July 2020 to April 2021 which initially enrolled 23 *H. pylori* infection positive patients with gastric cancer, 20 *H. pylori* infection negative patients with gastric cancer and 10 normal healthy participants as a control whose age ranged from 18-75 and had a clear enough awareness to sign an informed consent in this trial. However, 3 *H. pylori* infection positive patients with gastric cancer were excluded when they violated the inclusion criteria after being enrolled. The inclusion criteria of gastric cancer patients included the following: (1) be definitely diagnosed as Ⅲ-Ⅳ phase gastric cancer, (2) had clear *H. pylori* detection results, (3) no history of gastric cancer, abdominal operation and *H. pylori* infection, (4) had not used antibiotics for more than 3 months. The (2), (3) and (4) inclusion criteria also applied to healthy people. The eligible persons were excluded if they participated other clinical experiments within four weeks, had other major diseases including cardiovascular, cerebrovascular diseases or liver, kidney, thyroid dysfunction, pregnant and lactating women. The TNM staging criteria of gastric cancer relied on the 8^th^ edition of the International Union Against Cancer/American Joint Committee on Cancer TNM classification system while the testing of *H. pylori* was evaluated based on C14-urea breath test in corresponding hospitals. All of the participants were collected the gastric juice and faeces as experimental samples which were distributed into groups (three gastric cancer patients were excluded due to do not meet the criteria of trial after being enrolled). The gastric juice and faeces samples from gastric cancer patients with *H. pylori* infection positive were grouped in PG (Gastric juice from *H. pylori* infection positive gastric cancer patients) and PI (Intestinal faeces from *H. pylori* infection positive gastric cancer patients), respectively. The gastric juice and faeces samples from gastric cancer patients with *H. pylori* infection negative were grouped in NG (Gastric juice from *H. pylori* infection negative gastric cancer patients) and NI (Intestinal faeces from* H. pylori* infection negative gastric cancer patients), respectively. The gastric juice and faeces samples from remaining 10 healthy people were grouped in HG (Gastric juice from healthy people) and HI (Intestinal faeces from healthy people), respectively.

Patient samples and data were obtained with written informed consent in accordance with the ethics committee requirements at the participating institute and the Declaration of Helsinki. The present study was approved by the Ethics Committee of the Second Affiliated Hospital of Nanchang University and Second Affiliated Hospital of Shandong University (2020-079 and KYLL-2020KJP-0185, respectively).

### Gastric Juice and Faeces Samples Collection

The trial did not impose additional interventions on patients and healthy people. For gastric juice samples collecting, abrosia and ban smoking were performed for 8 hours before collecting. Inform volunteers sampling time in advance to relieve anxiety of volunteers to avoid abnormal results caused by mental pressure according to the recommendation of M. J. Tetel et al. 21. 3-4 ml gastric juice was collected into test tube from every patient and healthy people via drainage tube. All the samples were stored at -80 °C. For faeces samples collecting, natural defecation was performed. For those volunteers who were difficult to defecate, rectal swab could be used as auxiliary equipment. Faeces were collected about 3-4 ml and stored at -80 °C. The physical condition of the patients was continued to be watched by senior clinical doctors who will give timely feedback. Participants in the experiment had the freedom to withdraw at any time if they had any discomfort ableness.

### Bacterial Genomic DNA Extraction and High-Throughput Sequencing

Both of gastric juice and faeces samples were collected from patients or healthy people and stored at -80 °C. The bacterial DNA was extracted via genomic DNA kits (Tiangen Biotech Co., Ltd., Beijing, China) and bead beating method. The extracted DNA was tested its quality and concentration through the using of a spectrophotometer at 230 nm (A 230) and 260 nm (A 260) (Nanodrop; Thermo Fisher Scientific, Inc., Waltham, MA, USA). And then the V4 region of the 16S rRNA genes was amplified using designed primers (F, 5'-AYTGGGYDTAAAGNG-3' and R, 5'-TACNVGGGTATCTAATCC-3') for high-throughput sequencing analysis by Illumina Novaseq platform in Personalbio Co., Ltd., Shanghai, China.

The paired-end reads sequencing was processed by Illumina MiSeq platform which was saved in the form of FASTQ and based on Greengenes databases (Gene Bank accession number PRJNA777736 and PRJNA777730). The amplicon sequencing variants (ASVs) analysis was performed by DADA2. The α diversity analysis including Chao 1 and Shannon indexes and Component analysis were achieved by QIIME2 (2019.4) at different levels of classification which were presented in the histograms. The Veen diagram used the R software and pheatmap. Furthermore, the Beta diversity analysis containing Principal coordinates analysis (PCoA) was used QIIME2 (2019.4) and R software to acquire distance matrix.

### Data analysis

All data were presented as mean ± SD. The Statistical analyses used the Grapad Prism 8 (https://www.grapad.com) and SPSS 23.0 software (SPSS Inc., Chicago, IL, USA) by t-test and non-way ANOVA. The p<0.05 was considered as statistically significant.

## Results

### Participants

**Figure 1:**
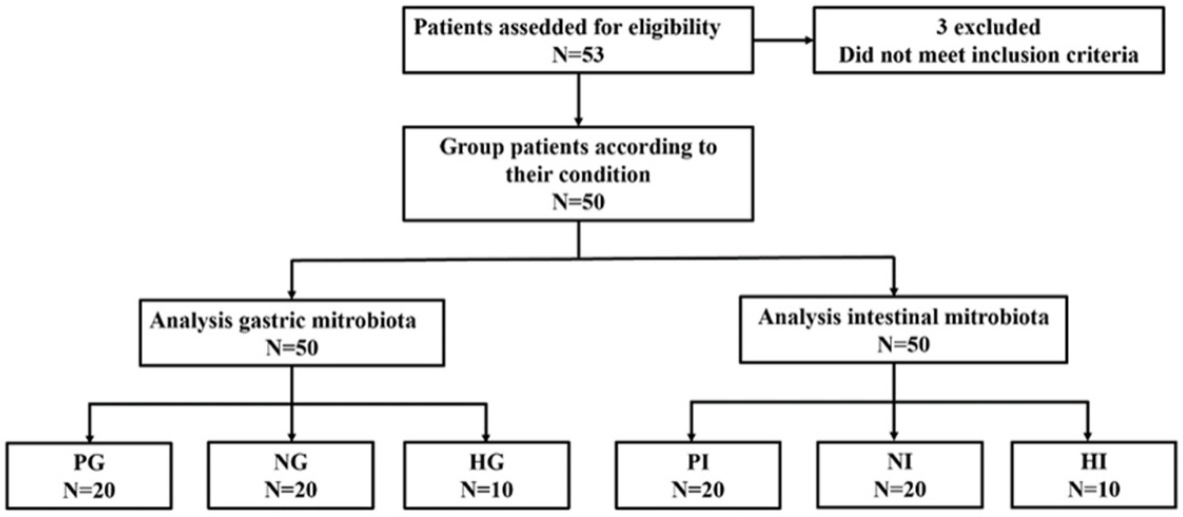
The trial enrolled 53 participants and 50 participants were selected finally which provide a total of 50 samples.

A total of 53 participants (29 male and 24 female) including 43 gastric cancer patients and 10 healthy people were enrolled in the trial. During the samples collection progress, 3 gastric cancer patients with *H. pylori* infection positive were excluded due to the treatment of probiotics after grouping. The flow diagram is shown in Figure 1. The results indicated that *H. pylori* infection was markedly correlated with dysplasia (P<0.05), intestinal metaplasia (P<0.05), and chronic atrophic gastritis (Table 1, p<0.05), and no significant difference were observed on clinical parameters age, gender, BMI, TNM stage, pathological type, tumor location and size (Table 2).

### The Alteration of Microbial Diversity and Richness in stomach

**Figure 2:**
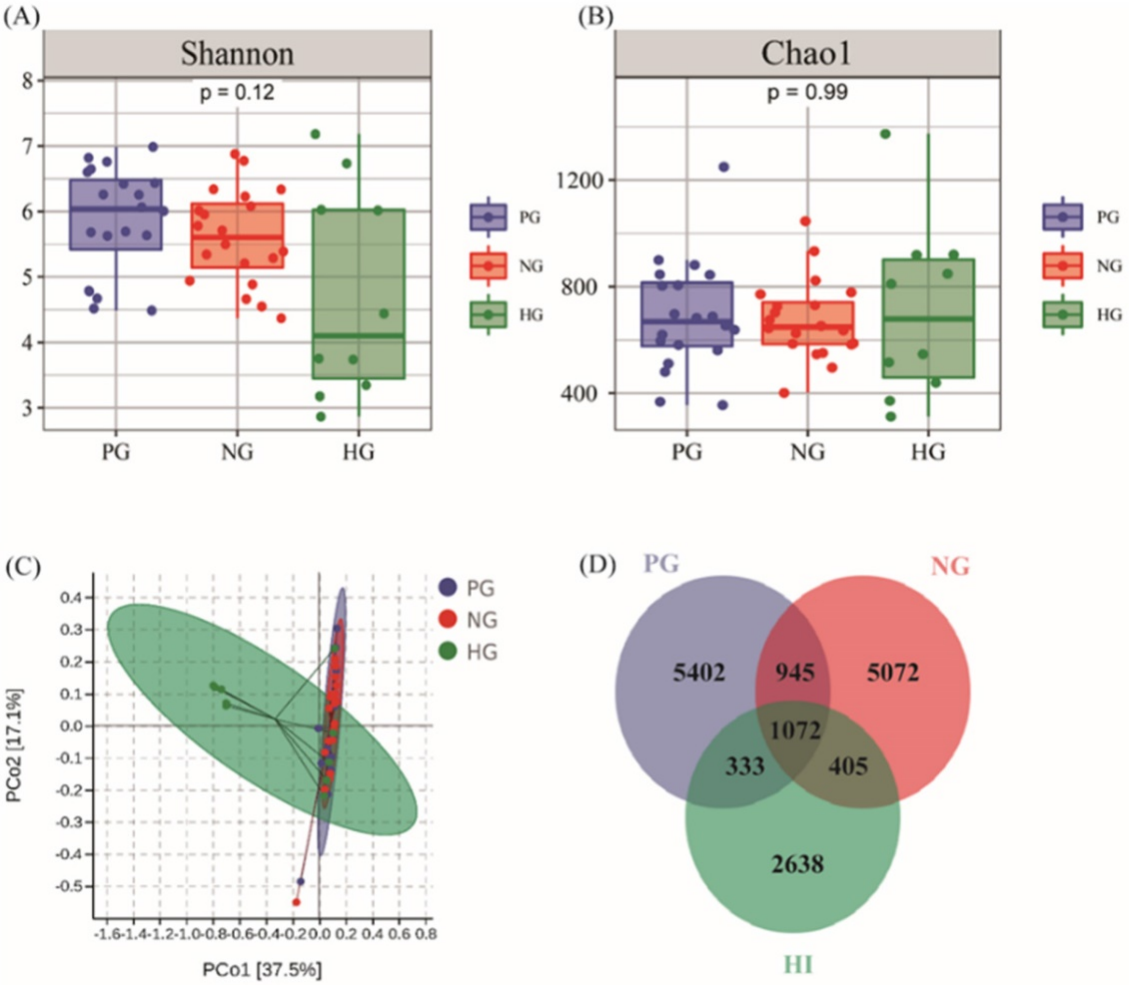
** The condition of richness and diversity in PG, NG and HG. (A)** Shannon analysis of gastric microbiota. **(B)** Chao 1 analysis of gastric microbiota. **(C)** PCoA analysis among PG (n=20), NG (n=20) and HG (n=10) groups. **(D)** Venn diagram of gastric bacteria species.

From the PG, NG and HG groups, a total of 33,829 OTUs were obtained from all samples with average of 676.59 OTUs per sample. The results from α diversity analysis seemed that the diversity and richness had no obvious difference in PG (Shannon: 5.86±0.80, Chao1: 688.87±200.68), NG (Shannon: 5.61±0.70, Chao1: 675.37±145.12) and HG (Shannon: 4.73±1.52, Chao1: 706.46±311.04) shown in Figure 2A and Figure 2B. Moreover, the results from principal coordinates analysis (PCoA) which was aimed to analysis the correlation among groups indicated that there was no huge separation in experimental groups while there was no significance observed between HG and experimental groups in Figure 2C. The Venn diagram results indicated that the number of 1,072 OTUs which accounted for 6.76% of bacteria were shared by all groups while the unique number of OTUs in PG, NG and HG groups was 5,402, 5,072 and 2,638, respectively (Figure 2D).

### The Composition of the Gastric Microbiota

**Figure 3:**
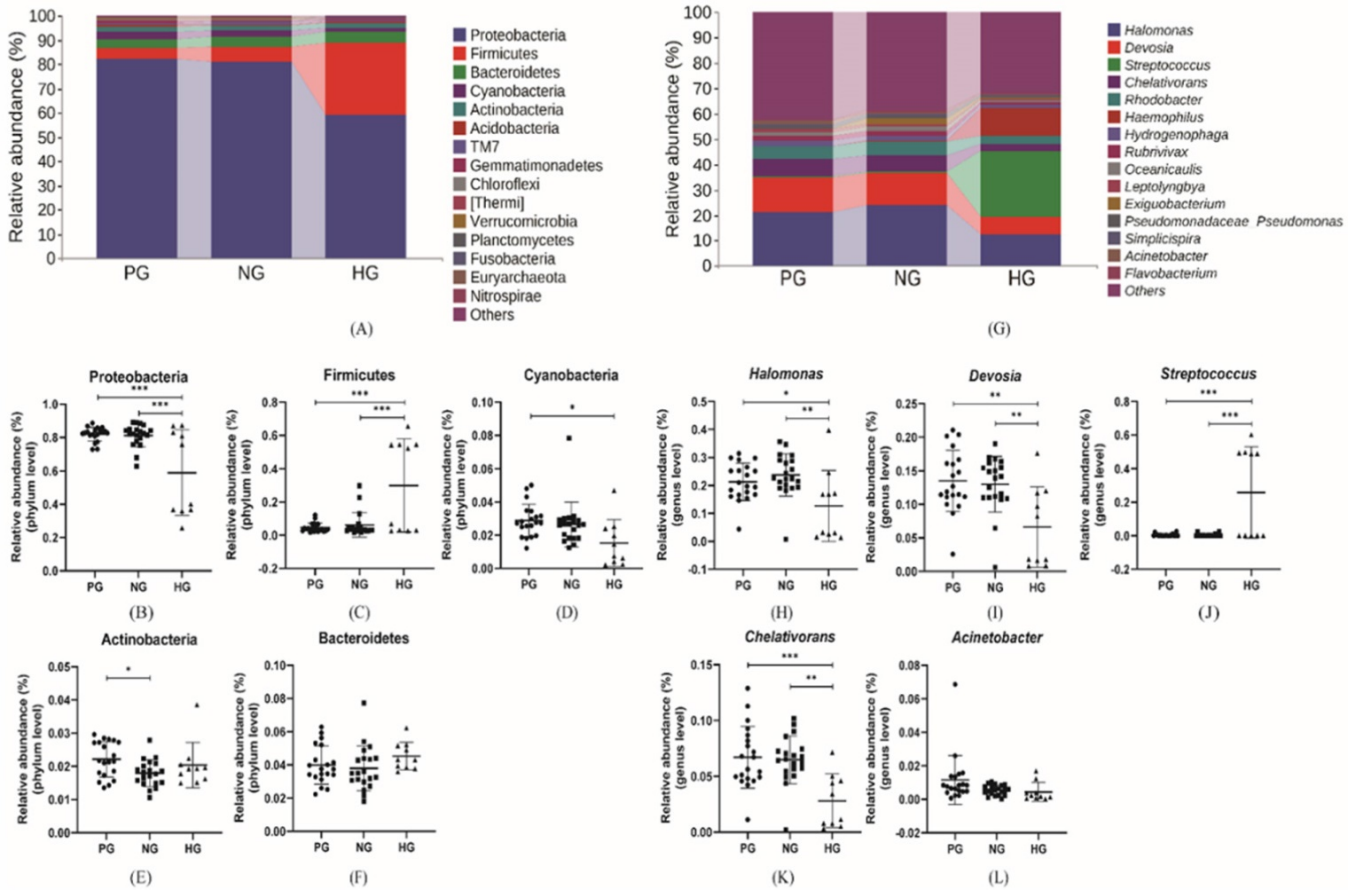
** (A), (G)** The relative abundance of comparatively higher bacteria at phylum and genus level. **(B-F)** The relative abundance of specific phylum bacteria. **(H-L)** The relative abundance of specific genus bacteria. *p<0.05, **p<0.01, ***p<0.005.

The composition and relative abundance of gastric microbiota was analyzed at phylum and genus level, shown in Figure 3. At the phylum level, all the groups shared the same dominant microbiota including Proteobacteria, Firmicutes, Bacteroidetes, Cyanobacteria and Actinobacteria which accounted for 0.965, 0.966 and 0.970 of the total analytic microbiotas in three groups respectively. In addition, the relative abundance of Proteobacteria (81.92%, 81.24% vs. 58.96%), Cyanobacteria (2.89%, 2.65% vs. 1.53%) in PG and NG groups were more abundant than HG group. Inversely, Firmicutes (4.59%, 6.19% vs. 29.94%) accounted much more in HG group rather than cancer groups. And the relative abundance of Bacteroidetes (3.99%, 3.79% vs. 4.53%) had no significant differences in all groups. The relative abundance of above dominant phyla in two cancer groups showed statistically significant difference only in Actinobacteria. At the genus level, the four most abundant genera were *Halomonas*, *Devosia*, *Streptococcus* and *Chelativorans*. The *Streptococcus* was the second most abundant genus in the HG group but was difficult to discover in both PG and NG groups, whose second most abundant genus was *Devosia*. The results indicated that in gastric cancer groups, in addition to *Streptococcus* (0.45%, 0.38% vs. 25.78%), the relative abundance of *Halomonas* (21.33%, 23.76% vs. 12.70%), *Devosia* (13.52%, 13.02% vs 6.63%), *Acinetobacter* (1.16%, 0.55% vs. 0.44%), and *Chelativorans* (6.70%, 6.78% vs. 2.81%) trended to increased compared with HG group.

### The Alteration of Microbial Diversity and Richness in Intestine

**Figure 4:**
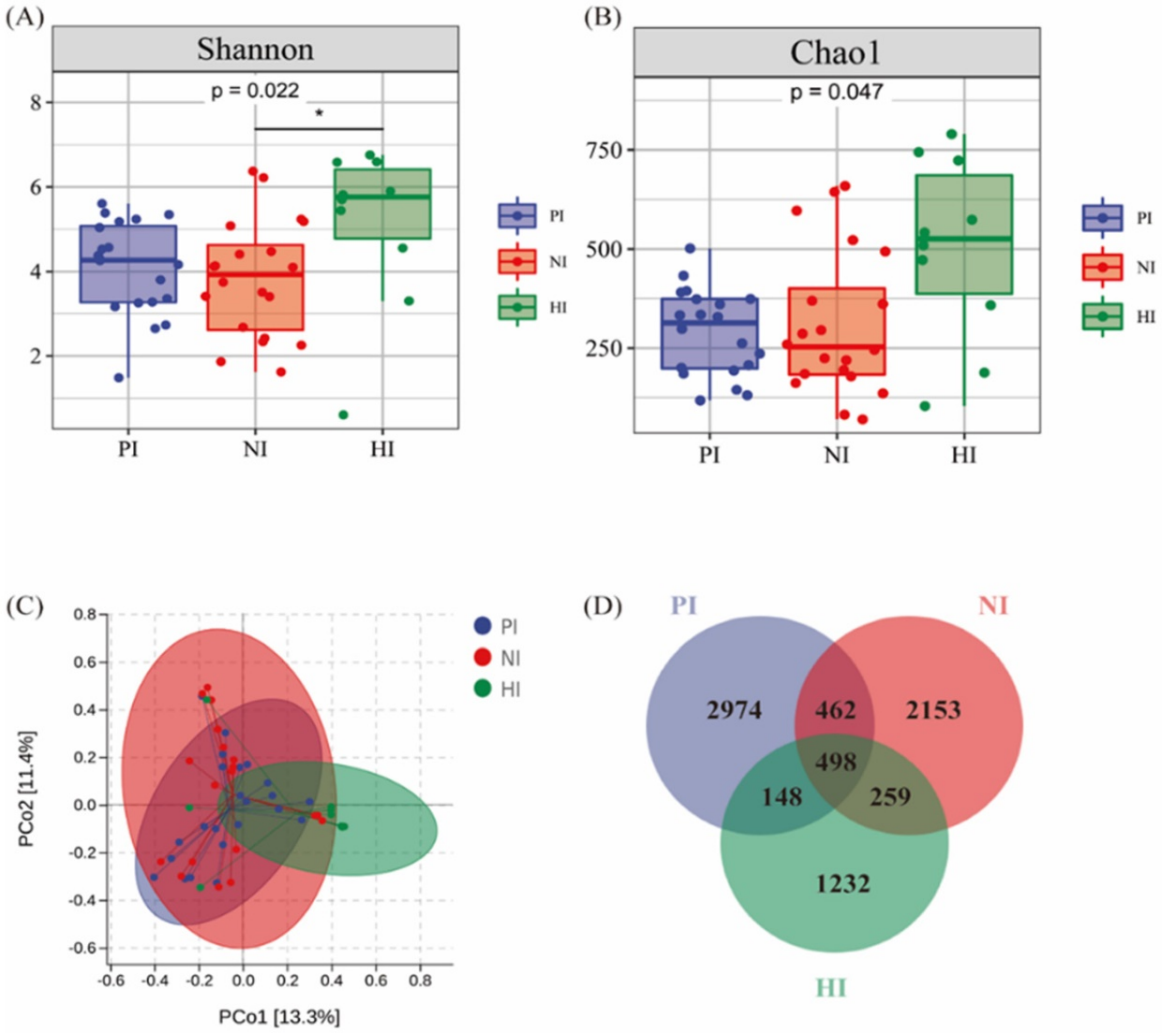
The alteration of Microbial Diversity and Richness in intestine. **(A)** Shannon analysis of gastric microbiota. **(B)** Chao 1 analysis of gastric microbiota. **(C)** PCoA analysis among PI (n=20), NI (n=20) and HI (n=10) groups. **(D)** Venn diagram of fecal bacteria species.

The same analytical method was approved in analysis of intestinal microbiota including a total of 16,443 OTUs with average of 328.88 OTUs per sample shown in Figure 4. Fifty fecal samples from PI, NI and HI groups, the Shannon index (4.08±1.07, 3.83±1.34 vs. 5.13±1.81) and Chao1 index (290.22±106.18, 309.45±201.40 vs. 500.67±218.14) shown in Figure 4A and Figure 4B which indicated that the diversity and richness of intestinal microbiota decreased in both gastric cancer groups (p<0.05). The PCoA diagram results indicated that there was presence of great difference of bacterial composition between cancer group and control group shown in Figure 4C. However, the HI and NI groups clustered together. The Venn diagram showed that there were 460 common OTUs sequenced in PI, NI and HI groups. Also the number of special OTUs in PI, NI and HI groups were 2,124, 2,324 and 1,279. In conclusion, the richness and diversity difference between gastric cancer groups and HI groups were more obvious than this difference in gastric microbiota. However, the effect of *H. pylori* was not fully demonstrated in PI and NI groups.

### The Composition of the Intestinal Microbiota

**Figure 5:**
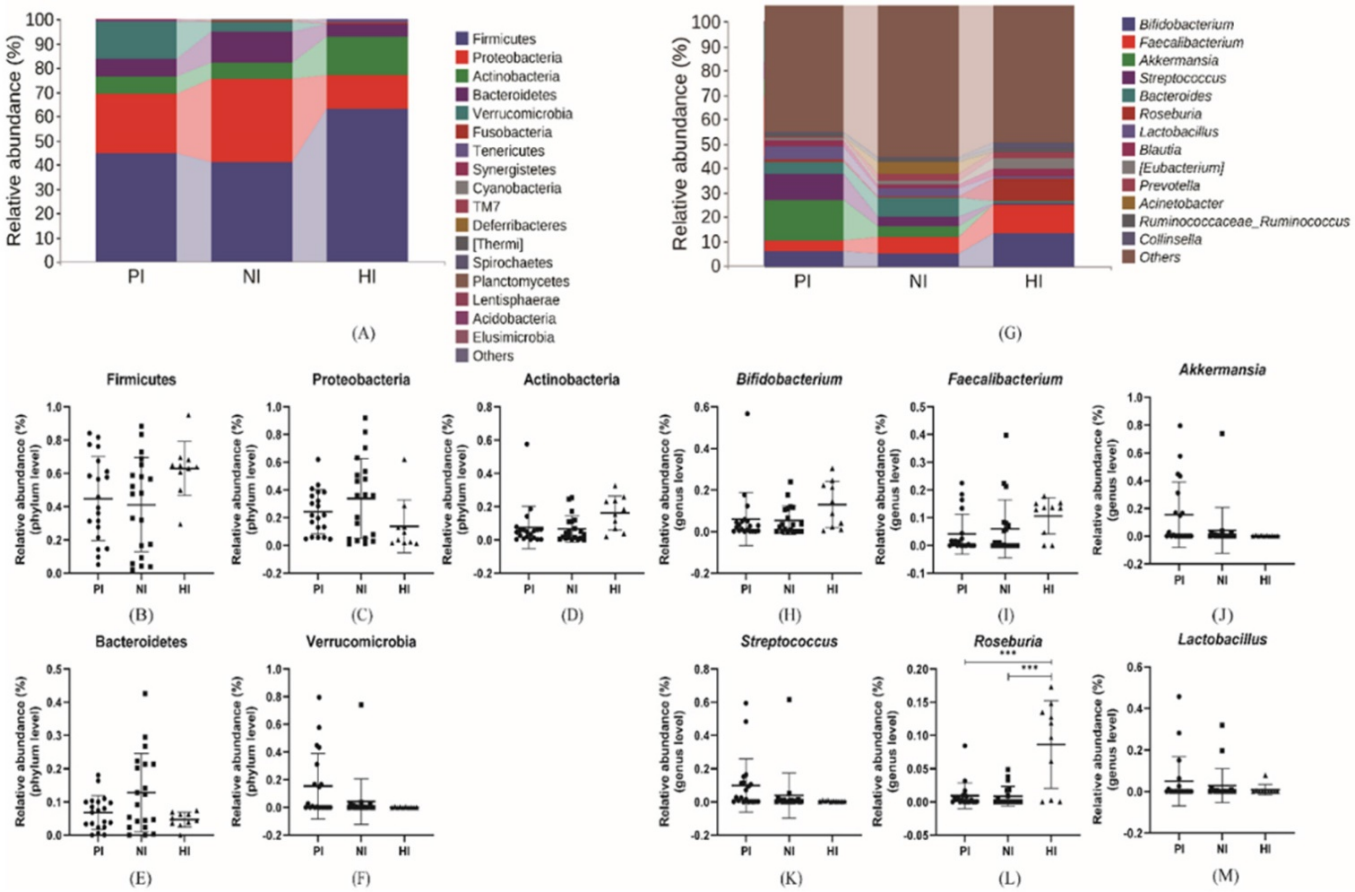
** (A), (G)** The relative abundance of comparatively higher bacteria at phylum and genus level. **(B-F)** The relative abundance of specific phylum bacteria. **(H-M)** The relative abundance of specific genus bacteria. *p<0.05, **p<0.01, ***p<0.005.

As same as above microbial composition and abundance analysis, the results analyzing from PI, NI and HI groups were shown in Figure 5 at phylum and genus level. Firmicutes (44.89%, 41.24% vs. 62.97%), Actinobacteria (7.48%, 6.70% vs. 16.28%), which decreased in gastric cancer groups while Proteobacteria (24.22%, 33.96% vs. 13.83%), Bacteroidetes (6.82%, 12.82% vs. 4.68%) and Verrucomicrobia (15.46%, 4.23% vs. 0.13%) which increased in gastric cancer groups and constituted the five most dominant bacterial species which accounted for more than 97% of all intestinal bacteria. At the genus level, there were differences in the dominant bacteria that made up each group. In PI group, top 5 microorganism populations were *Akkermansia*,* Streptococcus*, *Bifidobacterium*,* Lactobacillus* and *Bacteroides* while in NI group were *Bacteroides*, *Faecalibacterium*,* Bifidobacterium*, *Acinetobacter* and *Akkermansia*. However, in HI group, the relative abundance of *Akkermansia* (15.46%, 4.23% vs. 0.13%), *Streptococcus* (9.93%, 3.89% vs. 0.49%) and *Bacteroides* (4.60%, 6.90% vs. 1.02%) decreased sharply while the *Bifidobacterium* (6.06%, 5.41% and 13.01%), *Faecalibacterium* (4.17%, 6.03% vs. 10.69%) and *Roseburia* (0.93%, 0.85% vs. 8.63%) increased compared with other groups and constituted the three most dominant genus. Comparing PI and NI groups, the *Akkermansia* and *Streptococcus* could be regarded as mark species in the PI group.

## Discussion

Gastric cancer ranks fifth among all malignancies and has been identified as one of the leading causes of cancer-related deaths worldwide 1. In healthy stomach, the colonization of *H. pylori* is widely acknowledged as a risk factor for gastric cancer, which results in the alteration of the gastrointestinal microbiota and eventually causes gastrointestinal microbiota disorder. However, its role on the alteration of gastrointestinal microbiota in gastric cancer patients remains unclear. It is therefore extremely important to analyze the changes in the gastrointestinal microbiota between *H. pylori* positive patients and negative patients with gastric cancer 22.

The present study enrolled 53 participants, and 50 participants were finally used including 20 *H. pylori* positive patients with gastric cancer, 20 *H. pylori* negative patients with gastric cancer and 10 healthy participants, to analysis the difference of gastrointestinal microbiota abundance and composition. Our high-throughput sequencing results indicated that little change in α diversity and β diversity between positive and negative groups for *H. pylori* infection positive and negative groups. The difference of microbiota composition in the stomach and intestine were then examined. In the stomach, the increasing of Proteobacteria in PG group indicated the alteration from health condition to gastric gastritis and gastric intestinal metaplasia, which may progress to gastric cancer finally. And at the genus level, our results suggested that the *Acinetobacter* was found with a higher relative abundance in PG group than NG group, and its role in the stomach is similar to that of *H. pylori*, resulting in gastritis, hypergastrinemia, over-production of inflammatory cytokine and gastrin, which cause gastric cancer and even colorectal cancer 23, 24. Besides, as the most abundant genus, the *Halomonas* was discovered increasing in gastric cancer patients compared with healthy individuals and is more strongly associated with gastric cancer, but studies on its mechanisms are scarce and in our study the difference between *H. pylori* positive and negative patients with gastric cancer in our study was inconspicuous 25. Meanwhile, the changes of intestinal microbiota composition were also worth discussing here. Individual variation in intestinal microbiota was greater compared with gastric microflora, suggesting that intestinal microbiota was unstable. At the phylum level, the dominant bacterial composition (Firmicutes Bacteroidetes and Actinobacteria) was broadly similar with previous results except there were much more abundant Proteobacteria in both gastric cancer groups, which, like the gastric results, are likely caused by deterioration of the gastrointestinal environment 26. At the genus level, compositionally, *Bifidobacterium*, *Akkermansia*, *Streptococcus* and *Lactobacillus* were dominant and their effect in the carcinogenesis is relatively clear. The relative abundance of *Bifidobacterium* reduced in gastric cancer patients and much lower in almost all *H. pylori*-infected individuals. The *Bifidobacterium* plays a protective effect in the gastrointestinal tract by reducing the production of pro-inflammatory cytokines and inhibiting the methylation of cell DNA and the acetylation of histone. Therefore, a reduction in the number of* Bifidobacterium* contributes to carcinogenesis 27, 28. On the contrary, the increasing in *Streptococcus* and *Lactobacillus* was discovered in the PI and NI groups and was more pronounced in the PI group.* Streptococcus* and *Lactobacillus* are also called lactic acid bacteria (LAB) and regarded as danger signs associated with cancer for there was accumulating evidence which indicate that the generation of reactive oxygen species (ROS) *in vivo* could be promoted by LAB to damage DNA and then induce the mutation of gastrointestinal cells 29, 30. Besides, LAB contributes to epithelial mesenchymal transition (EMT), inducing a loss of cell polarity and producing angiogenic substances through reductive reactions 31, 32. Furthermore, the other LAB production lactic acid is thought to be part of the fuel and chemical pathways of cancer cells, including activating hypoxia-inducible factor-1 (HIF-1) to support gastric cancer 33. Thus, over-production of LAB promotes cancer development and progression 34. Besides, the relative abundance of *Akkermansia* also increased in PI group compared with NI group. Although *Akkermansia* may improve obese conditions, its colonization of the intestinal tract has the potential to cause inflammation and thus induce cancer 35. However, the effective role and function of human gastrointestinal microbiota in relation to cancer should continue to be studied.

## Conclusions

**Figure 6:**
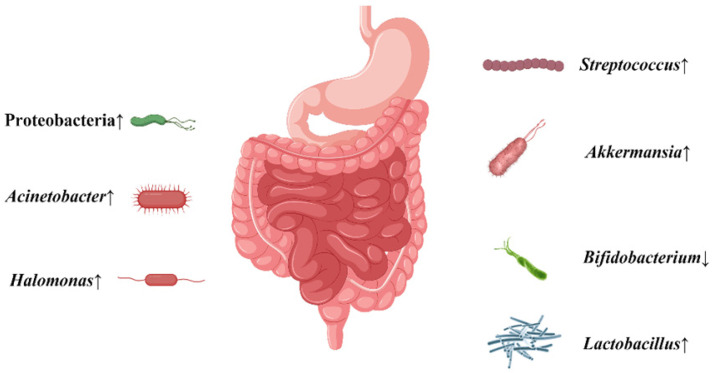
The conclusion about the alteration of gastrointestinal microbiota when gastric cancer patients were infected with H. pylori.

This clinical trial showed that the normal microbiota was disrupted, with increased levels of some bacteria thought to promote cancer, or decreased levels of some bacteria thought to prevent cancer in gastric patients which is more obvious with H. pylori infection (Figure 6). This may suggest that H. pylori infection may increase the possibility of gastric carcinogenesis and further development of gastric cancer via some disturbed gastrointestinal microbiota. This provides a new recommendation for the future prevention and treatment of gastric cancer. However, there are still many studies that need to be conducted before clinical application can be made 36. Considering the limited number in the present study, more cases are needed in our future work.

## Figures and Tables

**Table 1:** The relationship between *H. pylori* infection and pre-gastric cancer lesion in gastric cancer patients

	HP(+)	HP(-)	P-value
**Metastasis**			P>0.05
positive	10	11	
negative	10	9	
**Dysplasia**			P<0.05
positive	16	7	
negative	4	13	
**Intestinal metaplasia**			P<0.05
positive	17	10	
negative	3	10	
**Chronic atrophic gastritis**			P<0.05
positive	18	6	
negative	2	14	

**Table 2:** The basic data of participants

	HP(+)	HP(-)	P-value
**Sex**			P>0.05
male	14	16	
female	6	4	
**Age**			P>0.05
>45	12	14	
<45	8	6	
**TNM stage**			P>0.05
Ⅲ	15	13	
Ⅳ	5	7	
**Tumor location**			P>0.05
fundus	6	8	
body	5	6	
antrum	9	6	
**Tumor size**			P>0.05
<3 cm	12	10	
>3 cm	8	10	
**Pathological type**			P>0.05
well	13	14	
low	7	6	
**BMI**			P>0.05
<28	6	8	
>28	14	12	
